# Maternity and Mothering

**Published:** 1912-01-27

**Authors:** 


### MATERNITY AND MOTHERING.

(Inquiries and Correspondence are invited)

### Some Problems of Birth.

In a paper which he entitles "The Aristocracy of Birth" Dr. R. J. Ewart contributes to the *Child* many interesting facts and figures about the problems of birth as they affect medical officers of health. He protests, first of all, against the current methods of setting forth infant mortality; thus he pleads for such a statement as that nine hundred infants, out of a thousand born, reach the age of twelve months, rather than that the infant mortality for the first year is one hundred per thousand. There is, of course, no difference whatever in the fact stated by these two different ways; but Dr. Ewart argues that the change would denote a mental alteration and would be beneficial.

In determining the factors which together make up the claim of any given set of infants to be considered the rightful aristocracy of the race, the author necessarily deals very largely with rates and ratios of survival, and bases his conclusions upon them. Thus, he deals with the influence which the age of the mother has upon the chances of life of her child. Of 1,000 babies born before the mothers have completed their twentieth year, 830 will survive for twelve months; whereas of the same number whose mothers are twenty-eight years of age, over 900 will attain this age. After forty years the proportion of mothers who succeed in rearing a child for twelve months is only 600 out of 1,000. Moreover, these figures of survival are corroborated by consideration of the physical vigour and development of the children; for Kürosi has proved that the lustiest children are those born of mothers at or about the age of thirty.

It has long been familiar teaching that a woman's greatest chance of conception occurs between the ages of twenty and twenty-five. It has also been authoritatively pronounced (by Galton amongst others) that the second and third children of a family are, in the main, the strongest and healthiest. Of this latter fact, if fact it be, several explanations have been offered; and if they are valid, it may be that they explain also this effect of the mother's age upon the vigour of the child. For obviously the second and third children of women who marry (and are fruitful) under twenty-five may be expected to arrive not very far from the age of thirty.

A further curious point is noticed with regard to the sex of the children. Before the twenty-seventh year, mothers are more likely to bear girl children; and after that age to have boys. Thus under twenty years of age, mothers produce 1,120 girls to 1,000 boys; and after forty only a little over 500 girls to 1,000 boys. At intermediate ages the differences are not so great, and the dead point, as has been said, occurs at about twenty-seven. It is also said that the best girl, on the average, is born rather before the twenty-seventh year, and the best boy rather after it. The male excess is greatest during the months of January, February, and March, which Dr. Ewart regards as the true primeval breeding season for the human species.

A somewhat cognate subject, and one which also is mainly a question of statistical evidence, is that of the risk to life involved in the bearing of a child. The report of the Medical Officer to the Local Government Board for 1910-11, recently issued, supplies the latest available information on this subject. The number of deaths thus caused is still far too high in this country, though a gradual improvement is being brought about. It has been pointed out over and over again that the mortality rate from puerperal sepsis and other puerperal risks is disgracefully and quite unnecessarily high in this country. It is plain to everyone that such deaths from sepsis are deaths which could be and ought to be prevented altogether. Puerperal sepsis, therefore, ought to be steadily diminished by every factor which makes for the increased efficiency of the nation's midwives and medical men.

During the year to which the Local Government Board statistics refer (1909) there was a slight increase in the death-rate from puerperal sepsis; but it is satisfactory to know that notwithstanding this, the rate was lower than it has ever been, save in the immediately preceding year. The maternal mortality rate per 1,000 births in the period under review was 1.56 from puerperal sepsis, and 2.14 from other risks of pregnancy and childbirth; making a total of 3.7 per 1,000, as compared with 4.65 ten years ago. This reduction is a substantial one, but is still far from being satisfactory. The rate has diminished regularly, save for momentary checks in 1905 and 1909; and in respect of sepsis it has diminished more in proportion than in respect of accidents, some of which can hardly be described as preventible. Thus eclampsia, hyperemesis, and placenta prævia are unavoidable complications of childbirth, the death-rate from which will doubtless never be totally extinguished, though probably it will be materially diminished. But puerperal sepsis is quite a different matter, and its extinction or reduction to a negligible quantity is merely a matter of education and obstetric cleanliness.

The charts and diagrams which illustrate the report show very clearly the extraordinary variations between the statistics for different parts of the kingdom: and these differences, it would seem, are fairly constant from year to year. London has the lowest figure for puerperal accidents of any county in England and Wales, but an average one for sepsis.

The report further suggests that London's comparative superiority in respect of accidents in childbirth is due to the ease with which skilled assistance can be obtained both at hospitals and for private patients in all difficult and dangerous cases. Surely this should tell also in respect of the incidence of sepsis; London cannot be content with its present place on the list.

